# Impact of a Lifestyle Intervention on Gut Microbiome Composition: A Quasi-Controlled Before-and-After Analysis

**DOI:** 10.3390/metabo15110692

**Published:** 2025-10-24

**Authors:** Fatma Shehata, Karen M. Dwyer, Michael Axtens, Sean L. McGee, Leni R. Rivera

**Affiliations:** 1 School of Medicine, Institute for Mental and Physical Health and Clinical Translation, Deakin University, Geelong, VIC 3220, Australia; s222396088@deakin.edu.au (F.S.); sean.mcgee@deakin.edu.au (S.L.M.); 2 The Royal Melbourne Hospital, The University of Melbourne, Parkville, VIC 3050, Australia; karen.dwyer@mh.org.au; 3 School of Medicine, Faculty of Health, Deakin University, Waurn Ponds, Geelong, VIC 3220, Australia; michael.axtens@deakin.edu.au

**Keywords:** metabolic syndrome, gut microbiome, lifestyle intervention, 16S rRNA sequencing, metabolic health

## Abstract

**Background**: The human gastrointestinal tract harbors a complex microbiota that plays a vital role in metabolic health. Dysbiosis of the gut microbiome has been linked to metabolic syndrome (MetS), a growing health concern characterized by obesity, hypertension, and dyslipidemia, all of which are strongly associated with insulin resistance and low-grade inflammation. This study aimed to analyze changes in gut microbiome composition and metabolic parameters in individuals with MetS following a 3-month shared medical appointment program driven by a patient-centered agenda with an emphasis on lifestyle pillars of diet, activity, sleep, and stress management. **Methods**: Thirty-six individuals with MetS were recruited. Of these, 14 completed a structured metabolic health program with facilitated group appointments, including personalized dietary adjustments, increased physical activity, stress management, and clinical monitoring, while 22 served as an untreated group. Fecal samples were collected for full-length 16S rRNA sequencing. Clinical and biochemical parameters, including body weight, blood pressure, HbA1c, triglycerides, and liver enzymes, were assessed. Microbiome data were analyzed for alpha and beta diversity and differential abundance. Correlations between microbial genera and clinical parameters were evaluated using Spearman correlation. **Results**: Post-intervention, significant improvements were observed in body weight (*p* = 0.0061), HbA1c (*p* = 0.033), triglycerides (*p* = 0.047), AST (*p* = 0.016), and systolic blood pressure (*p* = 0.020). Alpha and beta diversity of the gut microbiome showed no significant changes. However, differential abundance analysis revealed increased levels of butyrate-producing and anti-inflammatory genera including Duncaniella, Megasphaera, Pseudoruminococcus, and Oliverpabstia. **Conclusions**: A 3-month lifestyle intervention in individuals with MetS was associated with marked improvements in metabolic health and beneficial shifts in gut microbiota composition. These findings suggest that even small lifestyle modifications may be a potential therapeutic target for metabolic syndrome management, highlighting the need for personalized approaches in future research.

## 1. Introduction

The gastrointestinal tract of humans contains a highly diverse and dynamic microbial community, the gut microbiota, which exerts considerable influence on the host under both healthy and disease states [1,2,3]. The gut microbiota contributes significantly to immune regulation, metabolic homeostasis, and protection against pathogenic organisms [4,5,6]. Disruptions in this microbial ecosystem, identified as gut dysbiosis, have been associated with the onset of various inflammatory conditions, obesity, and an increased risk of metabolic syndrome (MetS) [7,8,9]. This supports the Terrain theory, which proposes that an unhealthy internal environment can make the body more prone to disease [10].

Obesity has emerged as a critical global health issue, with its prevalence rising sharply across both developed and developing nations. In response, the World Health Organization has classified obesity as a worldwide health crisis and estimated that in 2022, 43% of adults aged 18 and older were classified as overweight, while 16% were diagnosed with obesity [11,12,13,14]. This trend is largely driven by urbanization, industrialization, and lifestyle shifts characterized by lower levels of physical activity and greater consumption of energy-rich, low-nutrient foods. In Australia, over two-thirds of adults are classified as overweight or obese, making this a particularly concerning issue. Obesity and MetS are deeply interconnected, as downstream consequences of common pathophysiological pathways and risk factors [15,16,17]. The global prevalence of MetS ranges from 12.5% to 31.4% [18]. In Australia, MetS affects around 30% of adults, with a continuous upward trend over the past two decades [19]. This rise mirrors shifts in dietary patterns, particularly the growing consumption of ultra-processed foods, and a decline in physical activity levels [20]. In a cohort of 41,146 individuals in Victoria, 12,228 (34%) had metabolic syndrome, meaning that approximately 1 in 3 individuals had MetS [21].

MetS is defined as a constellation of clinical conditions including abdominal obesity, systemic hypertension, dyslipidemia, and insulin resistance [22,23]. This syndrome is recognized as a precursor to multiple chronic diseases, such as cardiovascular disease and type 2 diabetes (T2D), and when combined with chronic kidney disease, it forms the cardiovascular–kidney–metabolic (CKM) syndrome [24,25].

MetS arises from the interaction of lifestyle, environmental, and genetic factors. Overnutrition and sedentary behavior promote excessive fat accumulation, particularly visceral adiposity, while socio-environmental factors such as stress, poor sleep, and socioeconomic disadvantage further contribute to weight gain and metabolic imbalance [26,27]. Genetic predisposition can also increase susceptibility to insulin resistance, obesity, dyslipidemia, or hypertension [28]. At the biological level, dysfunctional adipose tissue releases excess free fatty acids and inflammatory cytokines, which impair insulin signaling in muscle, liver, and adipose tissue, leading to insulin resistance [29]. To maintain glucose homeostasis, the pancreas compensates by increasing insulin secretion, resulting in hyperinsulinemia [30]. These disturbances give rise to the characteristic features of the syndrome: hypertension, due to sodium retention, sympathetic overactivity, and endothelial dysfunction; dyslipidemia, characterized by elevated triglycerides, reduced HDL cholesterol, and small dense LDL particles; and a prothrombotic state, with increased clotting factors and systemic inflammation [28,31]. Together, these abnormalities accelerate the development of type 2 diabetes, cardiovascular disease, and chronic kidney disease [28,32,33].

Evidence from previous studies suggests that lifestyle interventions, particularly those involving dietary improvements and increased physical activity, can significantly reduce the risk of obesity-related and MetS-associated diseases [34,35,36,37,38,39]. Indeed, the multi-center RCT Look AHEAD study compared the effect of a 12-year intensive lifestyle intervention with that of diabetes support and education on the incidence of kidney and cardiovascular disease. They showed that participants who attained remission from T2D had a 33% lower rate of CKD and 40% lower rate of CVD compared with those who did not, and the magnitude of risk reduction was greatest in those with longer remission [40].

However, whether these health benefits are associated with alterations to the microbiome remains poorly explored. Thus, the current study intended to characterize the gut microbiome composition in individuals with MetS and to evaluate the impact of a 3-month metabolic health program (MHP) centered on lifestyle modification on gut microbial profiles. We hypothesized that individuals with metabolic syndrome have reduced gut microbiome diversity and lower abundance of beneficial bacteria, and that participation in a lifestyle program would induce shifts in the gut microbiome alongside improvements in metabolic health.

## 2. Materials and Methods

### 2.1. Participants and Ethics Approval

Individuals with MetS were invited to join a metabolic health program of Shared Medical Appointments aimed to improve/reverse the features of MetS as previously described [41].

We used the National Heart, Lung, and Blood Institute (NHLBI) definition of MetS as a constellation of clinical conditions including abdominal obesity, systemic hypertension, dyslipidemia, and insulin resistance [42]. Exclusion criteria included participants who had used antibiotics in the previous three months, were taking medications affecting bowel function, had previously experienced gastrointestinal diseases (e.g., celiac disease, inflammatory bowel disease), or had undergone major gastrointestinal surgery (e.g., bariatric surgery, bowel resection). The study adhered to the STROBE guidelines for reporting observational studies of interventions and STROBE-ME for microbiome-related details [43]. Human ethics approval was received from the Deakin University Human Ethics Committee (2021-303).

### 2.2. Study Design

This was an implementation study with a quasi-controlled “within-subjects “ or “self-controlled” design. All participants (*n* = 36) were provided with personalized written information outlining the study objectives and inviting voluntary participation. All participants engaged in one-on-one consultations to determine baseline metabolic health. To account for potential regression to the mean and natural temporal variation, an untreated comparison group was included. This group comprised 22 individuals with metabolic syndrome who did not participate in the metabolic health program (MetS untreated). The remaining 14 participants engaged in shared medical appointments (6–8 participants per group). The dietary intervention recommended was personalized according to baseline metabolic testing and presence of insulin resistance. Priority was given to protein targets of at least 1 g/kg/day spread across meals. The remainder of the macronutrient composition—fat and carbohydrate—was tailored to individual needs and preferences. Given that insulin resistance underpins metabolic syndrome, lowering carbohydrate intake through eliminating ultra-processed foods and sugars was encouraged [44]. As part of the physical activity program, individualized plans based on capacity, emphasizing incremental progress (“something is better than nothing”) rather than rigid adherence to population-level targets that may discourage engagement, were promoted. Stress management strategies included guided mindfulness practice, and counseling on improved sleep practices was provided. Guidance on safe sun exposure for vitamin D attainment and targeted smoking cessation support was provided, and where appropriate, medications were deprescribed. These lifestyle components were designed to influence both metabolic health and gut microbiota composition, reflecting a holistic, patient-centered approach to managing MetS.

Individuals who participated in the metabolic health program (MHP) engaged in 6 visits over 3 months (week 1, 2, 4, 6, 10, and 14). They were reviewed again one-on-one at 12 months. Throughout the study, the physicians overseeing the participants managed clinical care, including medication adjustments, and arranged additional individual consultations as needed, in line with standard practice. Motivational interviewing techniques were used, enhanced by facilitated interaction between participants. The primary outcome measures were the changes from baseline to follow-up in the average values of metabolic syndrome components. Biophysical data were collected at the start and end of the program and included anthropometric (body weight), clinical (blood pressure), and biochemical data (blood glucose, glycated hemoglobin (HbA1c), triglycerides (TGs), high-density lipoprotein (HDL), uric acid, fasting insulin, urinary albumin-to-creatinine ratio (uACR), liver enzymes (aspartate aminotransferase “AST” and alanine aminotransferase “ALT”), and lipid subfraction analysis (LDL peak number and LDL pattern type), and biochemical measurements were conducted by a commercial pathology provider (Melbourne Pathology, Australia). For the intervention group, each participant served as their own control (before vs. after the program), while the MetS untreated group provided an additional comparison. Changes in biophysical markers of metabolic syndrome were analyzed in conjunction with changes in the microbiome.

### 2.3. Fecal Collection and Bacterial DNA Isolation and 16S rRNA Sequencing

At baseline, fecal samples were collected from all participants (*n* = 36). At the end of the program, follow-up fecal samples were obtained from the 14 participants who completed the intervention. Sample collection was conducted at home following a standardized protocol involving aseptic handling and collection in sterile tubes. Samples were stored at −80 °C until microbiome analysis. Bacterial DNA was extracted from the fecal samples using the QIAamp Fast DNA Stool Mini Kit, following the manufacturer’s protocol (Qiagen Pty Ltd., Dandenong, VIC, Australia). The extracted DNA (10 ng/μL) was then assessed for purity using a NanoDrop 1000 spectrophotometer (Thermo Scientific™, Wilmington, DE, USA). PCR amplification and full-length 16S rRNA sequencing were performed by the Australian Genome Research Facility (AGRF) according to its established protocol.

### 2.4. Statistical and Microbiota Data Analysis

Statistical analyses of continuous biophysical data were performed depending on their distribution. Normality of all continuous variables was assessed using the Shapiro–Wilk test. Variables that passed the normality test (*p* > 0.05) were analyzed using paired *t*-tests, while variables that did not follow a normal distribution or had small sample sizes were analyzed using the Wilcoxon matched-pairs test. Changes in categorical variables were evaluated using Fisher’s exact test, and descriptive statistics were reported for parameters with insufficient sample size for formal testing. Data are presented as mean ± standard deviation (SD). All analyses were conducted using GraphPad Prism (version 10.5.0).

Post-bioinformatics analyses of fecal microbiota were conducted for all participants using RStudio (version 2024.12.1 +563). Analyses included assessment of changes in relative abundance, alpha diversity, beta diversity, and differential abundance. Alpha diversity (Shannon and Fisher indices) was calculated to evaluate microbial diversity within each sample using the estimate_richness function in the phyloseq package and visualized with boxplots via phyloseq and ggplot2. Beta diversity was assessed using Bray–Curtis dissimilarities and visualized through principal coordinate analysis (PCoA) plots in phyloseq. To test for differences in microbial composition between groups, permutational multivariate analysis of variance (PERMANOVA) [45] was performed on the Bray–Curtis dissimilarity matrix using the adonis2 function in the vegan package [46]. Additionally, DESeq2 was applied to identify genera and species with differential abundance between samples, with significance defined at a false discovery rate (FDR) < 0.05, within the phyloseq framework [47]. Correlations between microbiota and clinical parameters were further examined. These analyses were restricted to a subset of 6 participants for whom complete microbiota and clinical data were available at both time points. Correlations between genus-level relative abundances and clinical variables were assessed using Spearman correlation analysis. Genus-level relative abundance values were extracted from the phyloseq object and correlated with clinical parameters (e.g., body weight, TGs, AST, SBP, HbA1c, etc.) using the cor.test function in R (2024-10-31). *p*-values from Spearman correlations were adjusted for multiple comparisons using the false discovery rate (FDR), with significance defined as FDR < 0.05. The resulting correlation coefficients and *p*-values were visualized as a heatmap using the pheatmap package in R.

## 3. Results

### 3.1. Clinical Parameters of the Participants

As certain clinical data were unavailable for some participants, the number of individuals included in each respective analysis is detailed in Table 1. The metabolic health program resulted in significant improvements across multiple domains. There was no significant difference in body weight between the untreated MetS group (94.9 ± 15.1 kg, *n* = 9) and the before-MHP group (85.2 ± 8.2 kg, *p* = 0.1330). However, body weight significantly decreased after MHP, from 85.2 ± 8.2 kg to 79.8 ± 8.6 kg (*n* = 6, *p* = 0.0061). Similarly, HbA1c did not differ significantly between the untreated MetS group (6.79 ± 1.93%, *n* = 13) and the before-MHP group (7.03 ± 1.02%, *p* = 0.2011). However, HbA1c significantly decreased after MHP, from 7.03 ± 1.02% to 6.30 ± 0.62% (*n* = 6, *p* = 0.0330). Triglyceride levels showed no significant difference between the untreated MetS group (1.78 ± 1.08 mmol/L) and the before-MHP group (2.23 ± 1.01 mmol/L, *p* = 0.7186). After the MHP, triglycerides significantly decreased from 2.23 ± 1.01 mmol/L to 1.43 ± 0.48 mmol/L (*n* = 6, *p* = 0.0472). AST levels were also similar between the untreated MetS group (24.0 ± 6.19 U/L, *n* = 6) and the before-MHP group (28.2 ± 7.1 U/L, *p* = 0.0.3040). However, AST significantly decreased after MHP, from 28.2 ± 7.1 U/L to 23.8 ± 7.2 U/L (*n* = 6, *p* = 0.0155).

Systolic blood pressure did not differ significantly between the untreated MetS group (150.3 ± 18.2 mmHg, *n* = 9) and the before-MHP group (160.7 ± 13.7 mmHg, *p* = 0.2331). However, it significantly declined after MHP, from 160.7 ± 13.7 mmHg to 139.3 ± 15.2 mmHg (*n* = 6, *p* = 0.0199).

ALT levels showed no significant difference between the untreated MetS group (33.5 ± 14.63 U/L, *n* = 6) and the before-MHP group (26.0 ± 10.8 U/L, *p* = 0.3385). ALT showed a decreasing trend after MHP to 21.3 ± 9.3 U/L (*n* = 6, *p* = 0.0696), though the change did not reach statistical significance. Similarly, there was no significant difference in uACR between the untreated MetS group (21.6 ± 28.86 mg/g, *n* = 7) and the before-MHP group (150.7 ± 366 mg/g, *p* = 0.3907). uACR declined after MHP to 125.8.7 ± 289.3 mg/g (*n* = 6, *p* = 0.0625), showing a trend towards improvement. For fasting insulin, statistical testing could not be performed due to the small sample size (*n* = 2). However, a notable decrease was observed after MHP, from 29.0 ± 4.2 µU/mL to 14.0 ± 4.2 µU/mL, compared with 16.83 ± 11.07 µU/mL in the untreated group (*n* = 8) (Table 1).

### 3.2. Effect of the Metabolic Health Program on Gut Microbiota

#### 3.2.1. Alpha Diversity

**Figure 1:**
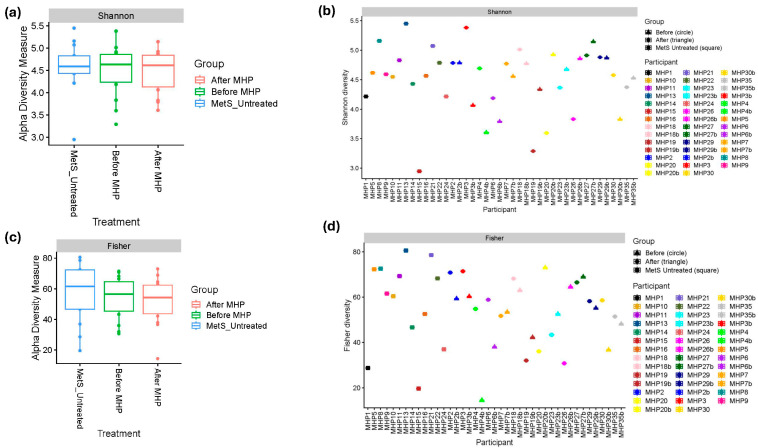
Alpha diversity grouped by treatment (untreated MetS, before MHP, and after MHP). (**a**,**b**) Shannon’s diversity index; (**c**,**d**) Fisher’s diversity index. The horizontal line inside each box indicates the median. Whiskers extend to the minimum and maximum values within 1.5 times the interquartile range (IQR) from the 1st and 3rd quartiles. The boxes represent the IQR, spanning from the 1st to the 3rd quartile. Solid dots (●) outside the whiskers indicate values exceeding 1.5 times but less than 3 times the IQR. Plots were created from raw, untrimmed data. Samples of MetS untreated participants are shown as squares, before MHP as circles, and after MHP as triangles.

Indices of alpha diversity were used to describe ecological similarities and dissimilarities within a microbiome community. Shannon diversity incorporates both the richness and evenness of species, while the Fisher index considers species richness. No statistically significant differences were observed among the groups: untreated MetS vs. before MHP (Shannon: *p* = 0.946; Fisher: *p* = 0.4532) and before vs. after MHP (Shannon: *p* = 0.95; Fisher: *p* = 1.00) (Figure 1a–d). Although individual variation was observed in the intervention group, alpha diversity appeared to decrease in six participants, increase in six participants, and remained unchanged in two following the program.

#### 3.2.2. Beta Diversity

**Figure 2:**
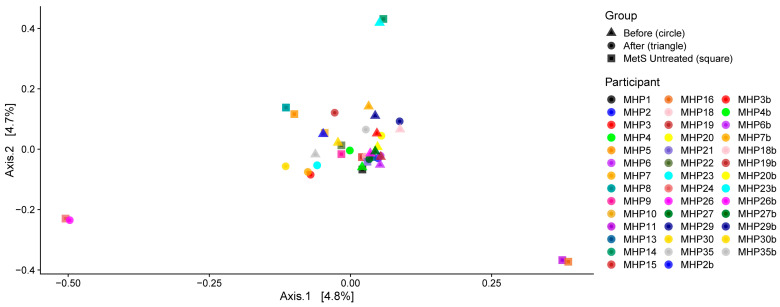
Principal coordinate analysis (PCoA)—beta diversity. Bray–Curtis, ordination plot showing the differences in gut microbiota composition from the MetS untreated group and intervention participants before and after the MHP. The microbiota profiles of intervention participants before and after the program are represented as follows: colors indicate individual participants, with matching colors for paired samples (e.g., participant MHP2 before and after are both blue), the MetS untreated group is also assigned distinct colors, separate from the intervention participants. Samples of MetS untreated participants are shown as squares, before MHP as circles, and after MHP as triangles.

Bray–Curtis was used to quantify similarities and differences between groups based on the composition and abundance of gut microbial community members, and a principal coordinate analysis (PCoA) plot was generated. No statistically significant differences in gut microbiota composition were observed among the groups: untreated MetS vs. before MHP (R^2^ = 0.040; *p* = 0.34) and before vs. after MHP (R^2^ = 0.037; *p* = 0.41) (Figure 2). Consistent with the findings for alpha diversity, individual-level variation was evident, with observable shifts in each intervention participant’s data before and after the program.

#### 3.2.3. Relative Abundance—Phylum Level

A visual comparison of microbiota profiles at the phylum level showed that the untreated MetS group had a relative abundance of Proteobacteria at 4.84%, compared to 6.55% before MHP and 11.39% after MHP. For Verrucomicrobiota, the untreated MetS group had 1.86%, compared to 2.24% before MHP and 2.69% after MHP. Similarly, Actinobacteriota was lowest in the untreated group (0.34%), 1.05% before MHP, and increased to 1.81% after MHP.

**Figure 3:**
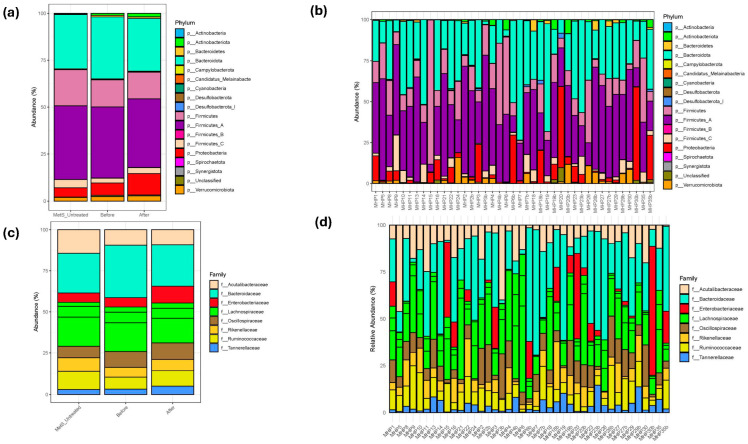
Relative abundance. Stacked bar charts showing the relative abundance of amplicon sequence variants (ASVs) at the phylum (**a**,**b**) and family (**c**,**d**) taxonomic levels. (**a**) Group-level data by treatment (untreated MetS, before MHP, and after MHP). (**b**) Paired data for each intervention participant and MetS untreated participants. (**c**) Family-level data grouped by treatment (before vs. after MHP) for intervention participants and MetS untreated participants; (**d**) Paired data per intervention participant and MetS untreated participants.

Within the Firmicutes phylum, now subdivided by the Genome Taxonomy Database (GTDB) to reflect more accurate phylogenetic relationships, the untreated MetS group had a relative abundance of Firmicutes_C at 4.41%, compared to 2.64% before MHP and 3.18% after MHP. Firmicutes_A, Firmicutes, and Firmicutes_B were 39.34%, 19.18%, and 0.07% in the untreated MetS group, compared to 37.87%, 14.48%, and 0.10% before MHP, and decreased after MHP to 36.63%, 14.26%, and 0.07%, respectively. Bacteroidota was 29.08% in the untreated MetS group, 33.05% before MHP, and 28.18% after MHP. Cyanobacteria was 0.12% in the untreated group, 0.08% before MHP, and 0.04% after MHP. Campylobacterota and Spirochaetota, absent before MHP, appeared after MHP at 0.01% each, compared to 0.004% and 0.02% in the untreated group. Synergistota was absent in the untreated group, 0.01% before MHP, and declined to 0.001% after MHP (Figure 3a,b).

In addition, a comparison of microbiota profiles revealed shifts in the relative abundance of several bacterial families following the MHP. At the family level, the untreated MetS group had Bacteroidaceae at 24.21%, compared to 31.95% before MHP and 25.23% after MHP. Lachnospiraceae was 26.85% in the untreated group, 27.08% before MHP, and decreased to 24.27% after MHP. Acutalibacteraceae was 14.29% in the untreated group, 9.4% before MHP, and 9.14% after MHP. In contrast, Enterobacteriaceae increased after MHP to 10.27%, compared to 5.67% before MHP and 5.63% in the untreated group. Ruminococcaceae was 11.04% in the untreated group, 7.27% before MHP, and 9.45% after MHP. Oscillospiraceae was 6.93% in the untreated group, 9.52% before MHP, and 10.1% after MHP. Rikenellaceae was 8.2% in the untreated group, 6.00% before MHP, and 6.7% after MHP. Finally, Tannerellaceae was 2.84% in the untreated group, 3.1% before MHP, and increased to 5.00% after MHP (Figure 3c,d).

#### 3.2.4. Differential Abundance Analysis

**Figure 4:**
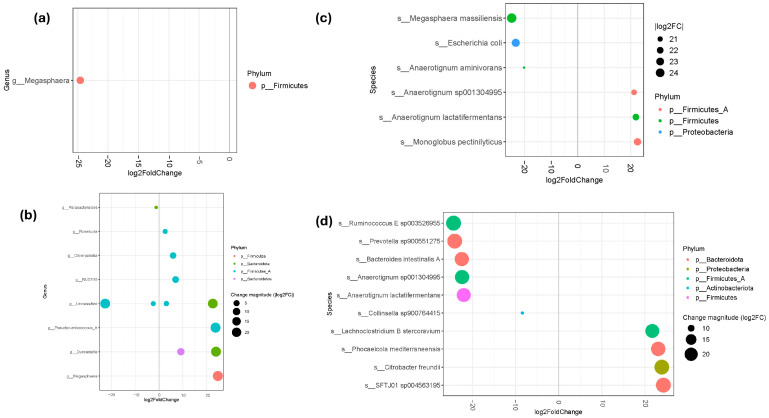
Key differentially abundant taxa. (**a**) Differentially abundant genera in the untreated MetS group and (**b**) in the after-MHP group; (**c**) Differentially abundant species in the untreated MetS group and (**d**) in the after-MHP group. Only genera and species that were significantly different in relative abundance (FDR ≤ 0.05) are shown in the plot, with log2 fold change determined using DESeq2. Dot size reflects the magnitude of change (|log2FC|), with larger dots indicating greater shifts in abundance.

In the untreated MetS group, Megasphaera abundance decreased (log2FC = −24.56) compared to the before-MHP group (Figure 4a). After the MHP, shifts in bacterial abundance were observed at the genus level across different phyla. Genera that significantly increased following the program included Duncaniella (log2FC = +23.86 and +9.13), Megasphaera (log2FC = +24.65), Pseudoruminococcus_A (log2FC = +23.66), RUG115 (log2FC = +6.93), Oliverpabstia (log2FC = +5.88), and Roseburia (log2FC = +2.55). In contrast, Parabacteroides showed a decrease in abundance (log2FC = −1.21) (Figure 4b).

Additionally, in the untreated MetS group, increases were observed in *Monoglobus pectinilyticus* (log2FC = +22.53), *Anaerotignum sp001304995* (log2FC = +21.24), and *Anaerotignum lactatifermentans* (log2FC = +21.92) compared to the before-MHP group (Figure 4c). After the MHP, we observed shifts in bacterial abundance at the species level across different phyla. Species that significantly increased following the program included *Phocaeicola mediterraneensis* (log2FC = +23.08), *Citrobacter freundii* (log2FC = +23.91), *SFTJ01 sp004563195* (log2FC = +24.29), and *Lachnoclostridium B stercoravium* (log2FC = +21.71) (Figure 4d).

In contrast, in the untreated group, decreases were observed in *Escherichia coli* (log2FC = −23.31), *Megasphaera massiliensis* (log2FC = −24.84), and *Anaerotignum aminivorans* (log2FC = −20.19) compared to the before-MHP group (Figure 4c). After the MHP, several species showed marked decreases in abundance, including *Bacteroides intestinalis A* (log2FC = −22.50), *Prevotella sp900551275* (log2FC = −24.16), *Collinsella sp900764415* (log2FC = −8.45), *Ruminococcus E sp003526955* (log2FC = −24.42), *Anaerotignum sp001304995* (log2FC = −22.45), and *Anaerotignum lactatifermentans* (log2FC = −22.05) (Figure 4d).

### 3.3. Correlation of Gut Microbiota with Clinical Parameters

**Figure 5:**
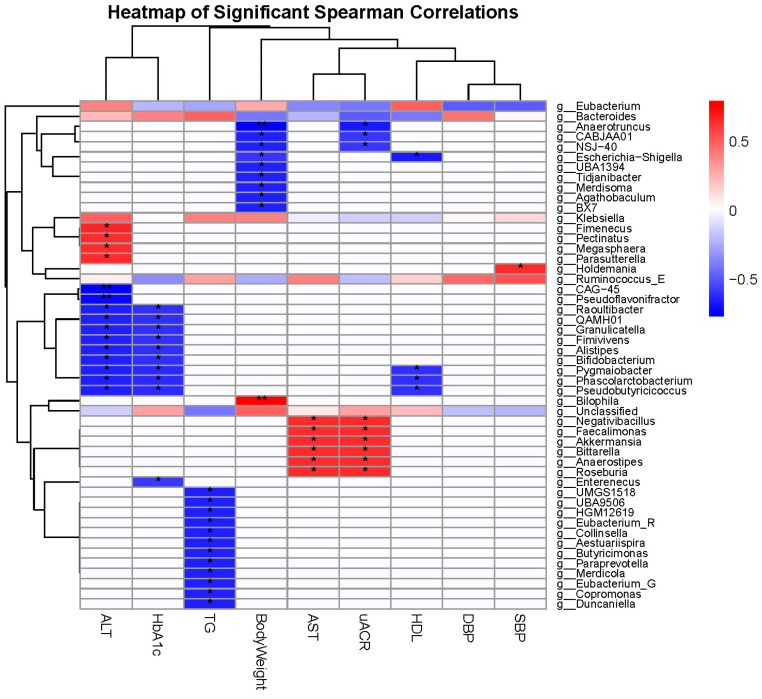
Correlation between gut microbiota and clinical parameters. Heatmap of Spearman correlations between bacterial genera (Y-axis) and clinical variables (systolic blood pressure (SBP), diastolic blood pressure (DBP), high-density lipoprotein (HDL), urinary albumin-to-creatinine ratio (uACR), aspartate aminotransferase (AST), body weight, triglycerides (TGs), glycated hemoglobin (HbA1c), and alanine aminotransferase (ALT)) (X-axis), annotated with log_2_ fold change values. Color key: Blue indicates negative correlations, white indicates no correlation, and red indicates positive correlations. The color scale ranges from –0.6 to 0.6, representing the strength and direction of Spearman correlation coefficients (Rho). Asterisks on the heatmap indicate significance levels based on Spearman correlation *p*-values: * *p* < 0.05, ** *p* < 0.01.

We assessed the correlation between gut microbial genera and a range of clinical parameters using Spearman correlation, revealing several genera with links to key metabolic health markers. For example, in relation to ALT, genera that were negatively correlated included CAG-45, Pseudoflavonifractor, Phascolarctobacterium, Pseudobutyricicoccus, Pygmaiobacter, Alistipes, Fimivivens, Granulicatella, QAMH01, and Raoultibacter (*p* = 0.0037–0.02, Rho = −0.77 to –0.66). In contrast, genera that were positively correlated included Fimenecus and Megasphaera (*p* = 0.0205–0.0234, Rho = 0.65 to 0.66) (Figure 5).

Relative to AST, genera that were positively correlated included Akkermansia, Bittarella, Faecalimonas, Negativibacillus, Anaerostipes, and Roseburia (*p* = 0.0214–0.024, Rho = 0.64 to 0.65). Concerning body weight, Bilophila showed the strongest positive correlation (*p* = 0.0021, Rho = 0.79), whereas Anaerotruncus, Agathobaculum, BX7, CABJAA01, NSJ-40, Escherichia-Shigella, Merdisoma, Tidjanibacter, and UBA1394 were negatively correlated (*p* = 0.02–0.04, Rho = −0.66 to–0.65) (Figure 5).

In terms of HbA1c, Phascolarctobacterium, Pseudobutyricicoccus, Pygmaiobacter, Enterenecus, Fimivivens, Granulicatella, QAMH01, and Raoultibacter were negatively correlated (*p* = 0.0304–0.0499, Rho = −0.62 to –0.58) (Figure 5).

In relation to HDL, Escherichia-Shigella, Phascolarctobacterium, Pseudobutyricicoccus, and Pygmaiobacter were negatively correlated (*p* = 0.0182–0.0411, Rho = −0.67 to −0.60). In relation to TGs, Merdicola, Paraprevotella, Copromonas, Duncaniella, Eubacterium_G, UBA9506, UMGS1518, and HGM12619 were negatively correlated (*p* = 0.0211–0.024, Rho = −0.65 to −0.64) (Figure 5).

Regarding SBP, only Holdemania was positively correlated (*p* = 0.0217, Rho = 0.65). In relation to uACR, Anaerotruncus was negatively correlated (*p* = 0.019, Rho = −0.66), whereas Akkermansia, Bittarella, Faecalimonas, Negativibacillus, Anaerostipes, and Roseburia were positively correlated (*p* = 0.022–0.025, Rho = 0.64 to 0.65) (Figure 5).

## 4. Discussion

Despite extensive research on the microbiome and metabolic disease, there are few studies that have investigated gut microbiome composition in individuals with MetS. The present study showed the effect of positive lifestyle changes on reshaping the gut microbiome composition in individuals with MetS.

Our results suggest that a 3-month positive lifestyle modification intervention in individuals with metabolic syndrome leads to significant improvements in some key clinical and biochemical parameters, including body weight, HbA1c, TGs, AST, and systolic blood pressure. These findings are consistent with previous reports on the benefits of lifestyle interventions in individuals with MetS [48,49,50], obesity [51], and T2D [52].

In our study, alpha diversity (Shannon and Fisher indices) and beta diversity (Bray–Curtis dissimilarity) were similar between untreated and pre-program groups and remained unchanged after the program, consistent with previous reports [53,54,55,56,57,58,59,60,61]. However, some prior reports contradict our findings, as they found that lifestyle modifications were associated with increased diversity [62,63]. These inconsistent findings on gut microbiome diversity suggest a need for further research, as microbiota composition is highly variable and influenced by factors such as diet, geography, ethnicity, genetics, physical activity, and medication use [64,65,66,67,68,69]. It is worth noting that the use of microbial diversity as a general marker of health is being reconsidered, with growing emphasis on specific health-associated taxa as potentially more precise and reliable markers of host health [70,71].

Unlike overall diversity measures, changes in the relative abundance of specific bacterial taxa were observed following the program, including enriched Duncaniella, Megasphaera, Pseudoruminococcus, and Oliverpabstia. Interestingly, Duncaniella has been found to be linked to carbohydrate metabolism and may influence gut health, energy balance, and immune function in the host, with particular emphasis on its anti-inflammatory and antioxidant activities [72,73,74,75,76].

Likewise, an increase in Megasphaera might be beneficial, as it is a butyrate producer that exerts beneficial effects on human health [77]. Notably, these SCFAs serve as a significant energy source for colonocytes [78] and help modulate the host’s energy balance by increasing nutrient availability [79]. In addition, Megasphaera has been associated with lower blood glucose levels, suggesting a protective mechanism against T2D [80,81,82,83,84], as well as lower blood lipids and improved hepatic steatosis [85]. Moreover, low Megasphaera abundance has been associated with diarrheal symptoms [86].

Intriguingly, enriched Pseudoruminococcus may benefit gut health through butyrate production and the degradation of complex carbohydrates [87,88,89]. Consistent with our findings, Zhang et al. detected a strong positive correlation between Pseudoruminococcus and β-alanine, a non-essential amino acid which may help ameliorate aspects of metabolic dysregulation associated with diabetes and related conditions [90,91,92]. Moreover, increased Oliverpabstia is likely to be advantageous, as it is known to produce major SCFAs, is involved in cobalamin (vitamin B12) biosynthesis, and has been associated with fat storage [93,94,95]. Another study observed a positive correlation between Oliverpabstia and defecation frequency [96]. Defecation frequency is considered as an important indicator of bowel function [97,98].

Moreover, our study revealed an increase in *Lachnoclostridium B stercoravium* in the after-MHP group, which might be favorable, as previous studies have reported that this species may be associated with the regulation of gut microbiota imbalance, reduction in intestinal permeability, and alleviation of inflammatory responses, thereby helping to prevent the progression and deterioration of non-alcoholic fatty liver disease [99]. Additionally, we observed a decrease in *Bacteroides intestinalis* in the after-MHP group, which might be beneficial, as previous studies have reported that this species is enriched in diarrhea-prone patients, where it promotes intestinal injury through the production of indole-3-acetate (IAA). IAA suppresses PI3K-Akt signaling, impairs epithelial renewal, and correlates with diarrhea severity, contributing to intestinal damage [100]. Furthermore, our results demonstrated that the increased abundance of *Anaerotignum lactatifermentans* in the untreated MetS group might be harmful, as this species has previously been reported to be enriched in individuals with high glucose levels and high BMI [101] and positively associated with plasma deoxycholic acid, a secondary bile acid whose elevated levels are linked to gut barrier disruption and inflammation [102].

The correlation between host clinical parameters and microbiota composition was further explored. The decrease in the genera Holdemania and Bilophila following the program may be advantageous, as they were positively associated with blood pressure and body weight. These results align with earlier reports, demonstrating that individuals with high BMI exhibit a higher abundance of Bilophila compared to lean subjects [103]. Additionally, Bilophila has been positively linked with body weight in individuals with obesity and metabolic syndrome [104], as well as in overweight children, where it correlates positively with BMI, hip circumference, and waist circumference [105]. Similarly, increased levels of Holdemania have been reported in hypertensive patients [106]. However, in hypertensive rat models, the relative abundance of Holdemania was negatively correlated with systolic blood pressure [107].

Likewise, the increase in the genera Paraprevotella and Phascolarctobacterium might be beneficial, as they have been linked to improvements in TG levels and HbA1c. This aligns with earlier research showing a negative correlation between Paraprevotella and TGs in obese rat models [108]. Similarly, Phascolarctobacterium was found to be negatively correlated with HbA1c in individuals with T2D [109], aligning with our findings. Interestingly, Phascolarctobacterium is known to produce SCFAs and play a beneficial role in host metabolic regulation [110].

In addition, we observed that the decrease in Akkermansia was associated with ameliorated AST levels. Consistent with previous studies, a positive correlation between Akkermansia abundance and AST was reported in individuals with autoimmune hepatitis [111]. Notably, Akkermansia can interact with Toll-like receptors (TLRs) [112,113,114,115,116,117], triggering the release of inflammatory cytokines, which may contribute to hepatocyte injury and elevated AST levels. Conversely, a negative correlation between Akkermansia and AST was observed in a metabolic-associated fatty liver disease mouse model [118]. These seemingly contradictory findings may reflect context-dependent effects, whereby Akkermansia’s interaction with TLRs and subsequent cytokine responses differ according to the underlying disease state or systemic immune environment.

A notable strength of our metabolic health program was its multifaceted design, integrating dietary modification, physical activity, stress management, and sleep improvement, delivered through a patient-centered, shared medical appointment format, which also fostered peer support [41]. While we observed changes in gut microbiome composition and metabolic parameters, these results reflect the effects of all program components. The existing literature suggests that each of these lifestyle factors can independently influence metabolic health and the gut microbiota. Dietary interventions, for example, can alter microbial composition and function [119]. Ren et al. reported that a three-month low-carbohydrate diet produced beneficial effects on glucometabolic parameters in individuals with T2DM [120]. In their study, both the intervention and control groups initially followed the same diet, but in the low-carbohydrate group, 150 g/day of carbohydrate foods was replaced with 56 g/day of almonds. After three months, both groups showed significant reductions in HbA1c; however, the low-carbohydrate group achieved a significantly lower level at the end of the study. Furthermore, the low-carbohydrate diet was associated with higher relative abundances of Roseburia, Ruminococcus, and Lactobacillus and a lower abundance of Eubacterium compared to the control diet. In addition, physical activity has been associated with increased microbial diversity [121]. In individuals with obesity, 8 weeks of moderate-to-high intensity exercise (2 to 4 times/week on 65% to 85% of heart rate reserve) improved insulin sensitivity and reduced visceral adiposity. Although gut microbiota alpha and beta diversity remained unchanged, the abundances of *Ruminococcus gauvreauii*, Lachnospiraceae, and Anaerostipes increased [122]. Nechalová et al. revealed the impact of a lifestyle modification program that included a calorie-restricted diet and at least 150 min of moderate-intensity physical activity per week for three months in adults with obesity. They observed a significant reduction in BMI, body fat percentage, waist circumference, hip circumference, and visceral fat after the program, along with a significant increase in the relative abundance of commensal bacteria (e.g., *Akkermansia muciniphila*, *Parabacteroides merdae*, and *Phocaeicola vulgatus*) and a significant decrease in the relative abundance of SCFA-producing bacteria (e.g., Butyrivibrio, Coprococcus, and Blautia) [123]. Stress reduction may help modulate inflammation-related dysbiosis [124]. A study on frontline healthcare workers exposed to COVID-19-related stress and exhibiting mental symptoms identified specific gut bacterial taxa linked to stress, including *[Eubacterium] eligens*, Bacteroides, Faecalibacterium, Streptococcus, Bifidobacterium, Sutterella, and Lachnospiraceae [125]. Moreover, improved sleep has been linked to healthier microbial profiles through the brain–gut–microbiota axis [126]. A study investigated gut microbiome composition and inflammatory markers in individuals with acute insomnia disorder (AID), chronic insomnia disorder (CID), and healthy controls (HCs). Compared to HCs, the CID group showed lower microbial richness, higher abundance of Actinobacteria, and increased levels of Blautia and *Eubacterium hallii*, while Faecalibacterium, Prevotella, and Roseburia were decreased. In contrast, the AID group exhibited reduced Firmicutes and Lachnospira and increased Bacteroides compared to HCs. Both the AID and CID groups had elevated IL-1β levels, and the CID group additionally had higher plasma TNF-α and IL-6 than the AID group and HCs [127]. Moreover, the gut microbiome can be altered by medication use. For example, in healthy individuals, taking up to 1 g of metformin (a diabetes medication) twice daily for 6 weeks altered gut microbiota composition, decreasing Intestinibacter and Clostridium while increasing Escherichia/Shigella and Bilophila wadsworthia. This was accompanied by an increase in body fat percentage and waist/hip ratio, a decrease in HbA1c, and fluctuating plasma B12 and cholesterol levels [128]. However, as all components were implemented simultaneously, our study cannot, and was not designed to, disentangle the relative contribution of each factor, and the observed changes should be interpreted as the result of the integrated program rather than any single intervention.

Another strength of our study was the use of full-length 16S rRNA sequencing, which offers enhanced taxonomic resolution by covering all nine hypervariable regions, enabling species-level identification and improved phylogenetic accuracy. This approach lays the groundwork for future studies to explore mechanistic links between specific microbial species and metabolic health outcomes, for example, by investigating their influence on host metabolism, inflammation, or energy balance.

A limitation of our study was the small number of participants (*n* = 14) and the short duration of the intervention (3 months), which may have limited the statistical power and generalizability of the findings. Importantly, correlation analyses were restricted to participants with complete paired samples (*n* = 14), which further constrained the robustness of the observed associations, even though all *p*-values were adjusted for multiple comparisons. The multifaceted nature of the intervention, along with variability in individual emphasis on lifestyle change, may have influenced the outcomes and contributed to inter-individual differences in microbiome shifts. Individual variability in microbial responses, potentially influenced by baseline metabolic status, lifestyle, or genetics, may also have affected the magnitude and direction of observed changes. Moreover, while we observed associations between specific microbial taxa and clinical markers, the underlying biological pathways linking these changes were not explored, and elucidating these mechanisms would require targeted functional studies. While we observed associations between the intervention and changes in the gut microbiome and clinical parameters, causality cannot be definitively established.

## 5. Conclusions

In summary, this study demonstrates that a 3-month lifestyle modification program in a facilitated group setting can lead to significant improvements in key clinical and biochemical parameters in individuals with metabolic syndrome. It also reveals significant shifts in microbiome composition following positive lifestyle changes, reinforcing lifestyle modification as a promising target for managing metabolic syndrome and related conditions. However, future studies should investigate the long-term effects of healthy lifestyle interventions on gut microbiome structure, using larger and more diverse cohorts. Importantly, the findings underscore the need for a personalized approach in microbiome research, as individual variability in microbial responses and lifestyle adherence may significantly influence outcomes.

## Figures and Tables

**Table 1:** Clinical and biochemical parameters of MetS untreated and intervention participants (before and after the MHP).

Parameter	MetS Untreated (Mean ± SD)	Before MHP (Mean ± SD)	After MHP (Mean ± SD)	*p*-Value (MetS Untreated vs. Before MHP)	*p*-Value (Before vs. After MHP)
Body Weight (kg)	94.89 ± 15.11	85.2 ± 8.2	79.8 ± 8.6	0.1330	0.0061 **
Blood Glucose (mmol/L)	5.58 ± 0.95	7.75 ± 2.66	6.70 ± 2.23	0.2011	0.2235
HbA1c (%)	6.79 ± 1.93	7.03 ± 1.02	6.30 ± 0.62	0.7186	0.0330 *
TGs (mmol/L)	1.783 ± 1.08	2.23 ± 1.01	1.43 ± 0.48	0.4021	0.0472 *
HDL (mmol/L)	1.39 ± 0.36	1.12 ± 0.17	1.30 ± 0.47	0.0462 *	0.3630
Uric Acid (mmol/L)	0.41 ± 0.11	0.41 ± 0.05	0.40 ± 0.06	>0.9999	>0.9999
Fasting Insulin (µU/mL)	16.83 ± 11.07	29.0 ± 4.2	14.0 ± 4.2	–	–
uACR (mg/mmol)	21.60 ± 28.86	150.7 ± 366	125.8 ± 289.3	0.3907	0.0625
AST (U/L)	24 ± 6.19	28.2 ± 7.1	23.8 ± 7.2	0.3040	0.0155 *
ALT (U/L)	33.50 ± 14.63	26.0 ± 10.8	21.3 ± 9.3	0.3385	0.0696
Systolic BP (mmHg)	150.3 ± 18.23	160.7 ± 13.7	139.3 ± 15.2	0.2331	0.0199 *
Diastolic BP (mmHg)	75.22 ± 11.32	85.0 ± 7.1	77.7 ± 10.3	0.0603	0.2052
LDL Peak Number	1.55 ± 0.82	2.4 ± 1.14	2.2 ± 0.84	0.1825	0.7489
LDL Pattern Type	Categorical change	Categorical change	Categorical change	0.3846	0.1

Data are presented as mean ± standard deviation (SD). *p*-values were calculated for comparisons between the MetS untreated group and the after-MHP group and within the intervention group (before vs. after MHP). Significance levels are indicated as follows: * *p* < 0.05 (significant), ** *p* < 0.01 (very significant). “–” indicates that statistical testing was not performed due to limited sample size. uACR: urinary albumin-to-creatinine ratio; AST: aspartate aminotransferase; ALT: alanine aminotransferase; BP: blood pressure; TGs: triglycerides; HDL: high-density lipoprotein; LDL: low-density lipoprotein.

## Data Availability

The original contributions presented in this study are included in the article. Further inquiries can be directed to the corresponding author.
